# Anesthetic Management for Dental Extraction in a Patient With Catecholaminergic Polymorphic Ventricular Tachycardia Syndrome: A Case Report

**DOI:** 10.7759/cureus.47224

**Published:** 2023-10-17

**Authors:** Edmund Wang, Van N Trinh, Bradlee J Bachar, Hussam Nagm

**Affiliations:** 1 Anesthesiology, Kaweah Health Medical Center, Visalia, USA; 2 Cardiac Anesthesiology, Kaweah Health Medical Center, Visalia, USA

**Keywords:** polymorphic ventricular tachycrdia, deep extubation, pediatric dental extraction, dexmedetomidine, life threatening arrhythmia, cpvt, cardiac anesthesiology, general anesthesiology, pediatric anesthesiology, catecholaminergic polymorphic ventricular tachycardia

## Abstract

Catecholaminergic polymorphic ventricular tachycardia (CPVT) is an inherited genetic disorder that predisposes patients to potentially fatal arrhythmia when under physical or emotional stress. Anesthetic management of patients with CPVT poses a huge challenge as physical and emotional stressors are common in the operating room. Stressors, such as pain and anxiety, must be carefully controlled to prevent life-threatening tachyarrhythmias. Currently there is a paucity of data on the anesthetic management of patients with CPVT. Herein, we present the anesthetic management that was implemented to ensure a safe perioperative course for a 16-year-old male with a history of CPVT who underwent dental extraction under general anesthesia.

## Introduction

Catecholaminergic polymorphic ventricular tachycardia (CPVT) was first described in 1975 as bidirectional ventricular tachycardia. It was noted to be a condition where tachyarrhythmia was precipitated by physical or emotional stress without any findings of structural abnormality in the heart [1]. The significance of the syndrome cannot be understated as untreated CPVT is a malignant syndrome with more than 50% of arrhythmic events and up to 25% of fatal or near-fatal cardiac events at 8 years follow-up [2]. The prevalence of CPVT is estimated to be about 1 in 10,000 people [3]. Furthermore, there is a clear correlation between the age of the first syncope and the severity of the disease, with a worse prognosis in the case of early occurrence. Symptoms include polymorphic ventricular tachycardia reproducibly induced during exercise tests, isoproterenol infusion, or emotional stress. CPVT can even cause sudden death and may be the first presenting symptom of the disease in children and adolescents [3]. Anesthetic management for CPVT poses a unique challenge as the perioperative surgical experience can be both emotionally and physically demanding for the patients. Stressors, such as venipuncture for intravenous access or postoperative pain and nausea are sufficient to induce dangerous arrhythmias in these patients [4,5]. Literature on the preparation and management of patients with CPVT is currently lacking. In this case report, we present a 16-year-old male with CPVT who underwent general anesthesia for dental extraction, with emphasis on the perioperative anesthetic management that was performed to preclude arrhythmias from occurring.

## Case presentation

**Figure 1:**
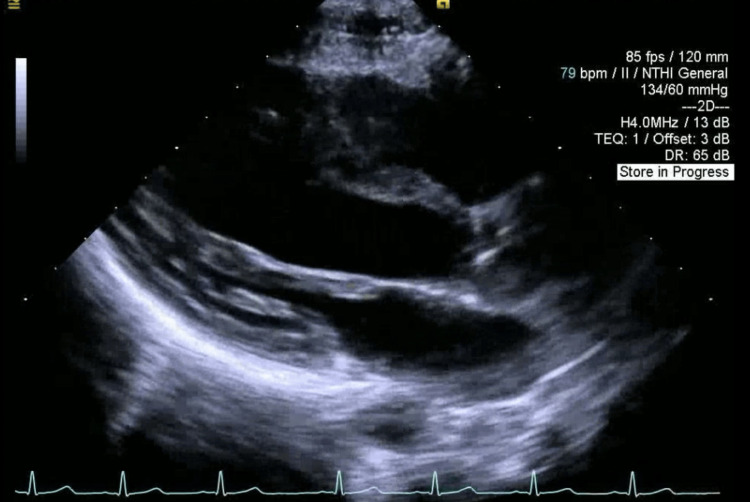
Transthoracic echocardiogram, parasternal long-axis view showing normal cardiac structure

**Figure 2:**
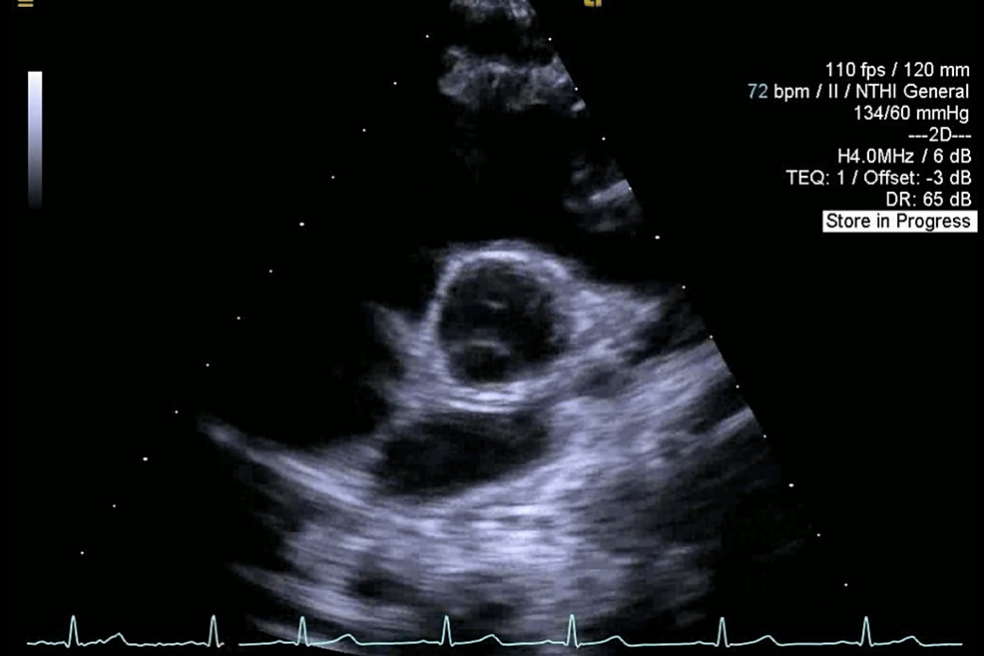
Transthoracic echocardiogram, parasternal short-axis aortic valve level view showing normal cardiac structure

A 16-year-old male (64 kg, BMI 19.9) presented for elective same-day surgery at our medical center. He had a past medical history of Ehlers-Danlos syndrome, surgical correction for pectus excavatum two years prior, and CPVT diagnosed with Holter monitor at age 13 after experiencing intermittent dizziness, tachycardia, and near-syncopal episodes. Prior cardiac workup included a pediatric echocardiogram that showed normal heart structure (Figure 1, 2) and function (Video 1).

**Video 1:** Transthoracic parasternal short-axis view showing normal cardiac function

**Figure 3:**
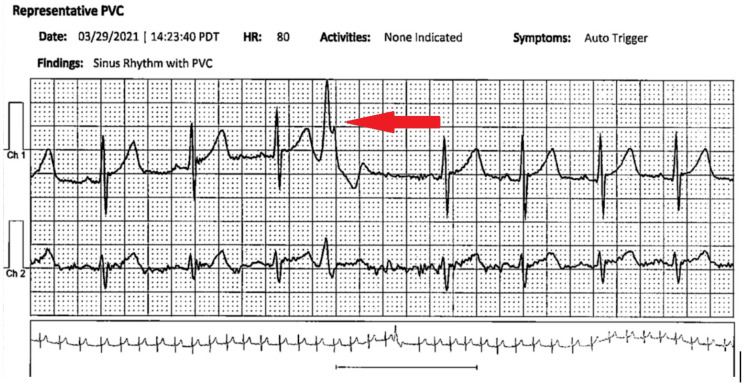
A rhythm strip from an 18-day Holter monitor showing a PVC PVC: premature ventricular contraction

The patient was initiated on nadolol 60 mg daily. To assess the therapeutic effect, a Holter monitor study was again performed which showed less than one percent premature ventricular contractions (PVCs) without any instances of supraventricular tachycardia, sinus pauses, or ventricular tachycardia (Figure 3). Although the placement of an automated implanted cardiac defibrillator (ICD) is a possible treatment for CPVT, it was deferred since his symptoms were resolved with beta-blockers alone. 

**Figure 4:**
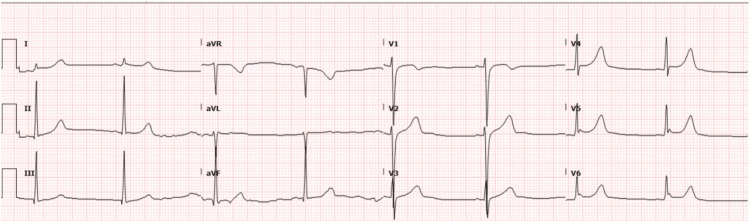
Preoperative 12-lead EKG showing sinus bradycardia of 49 bpm bpm: beats per minute

Our approach to this patient with CPVT centered around limiting emotional and physical stressors. On the day of surgery, the patient arrived at the hospital having fasted for more than 8 hours and took his usual morning dose of nadolol. A 20-gauge peripheral intravenous catheter was placed by an experienced provider. Vital signs were normal with a heart rate of 54 beats per minute (bpm), blood pressure of 110/52 mmHg, and a respiratory rate of 18 breaths per minute. Preoperative EKG showed sinus bradycardia at a rate of 49 bpm (Figure 4). The patient was premedicated with midazolam 2 mg in the preoperative area to alleviate anxiety and fears prior to transport to the operating room.

Upon entrance into the operating room, cardiac defibrillator pads were placed on the patient prophylactically to treat any potential stress-induced arrhythmias during surgery. The patient received fentanyl 100 mcg as additional premedication once he was moved onto the operating table. Once standard ASA monitors were applied, the patient was induced with propofol 200 mg and rocuronium 80 mg. Lidocaine 80 mg was given ahead of propofol to blunt the sympathetic response to laryngoscopy. Esmolol 10 mg was administered shortly after induction due to an increase in heart rate from 58 to 99 bpm following induction. Once the heart rate decreased to 66 bpm, direct laryngoscopy was performed. The patient was intubated with a 7.0 mm cuffed endotracheal tube without any complications and the heart rate remained stable.

After a discussion with the surgeon, we elected to proceed with plain lidocaine without epinephrine for surgical site infiltration. Although the additive epinephrine has beneficial vasoconstrictive effects which may help reduce bleeding and improve visualization of the surgical field, it was not utilized during this procedure due to the potential risk of tachycardia from catecholamine absorption into the bloodstream [6]. Maintenance of anesthesia was achieved with sevoflurane and dexmedetomidine (0.4 mcg/kg/hr without a loading bolus). Four impacted third molars (#1,16,17,32) and four bicuspids (#5,12,21,28) were extracted without any issues. Heart rate peaked at 75 bpm but gradually decreased to approximately 50 bpm throughout the procedure. For post-operative pain control, the surgeon performed bilateral superior and inferior alveolar nerve blocks and buccal infiltrations with lidocaine.

During the emergence of anesthesia, neuromuscular blockade was reversed with sugammadex. Dexamethasone 4 mg and ondansetron 4 mg were given for postoperative nausea and vomiting (PONV) prophylaxis. The patient was breathing spontaneously at a rate of 20 breaths per minute, therefore additional doses of fentanyl were administered until the patient was breathing at a rate of 12 breaths per minute and a deep extubation was executed without any complication to airway or hemodynamics. Supplemental oxygen via face mask was applied and the patient was transported to the post-anesthesia recovery unit (PACU). We remained with the patient at the bedside until he was awake and stable. Pain level was at a maximum of 3/10. No tachyarrhythmia was induced throughout the perioperative period. The patient was observed for an hour and after meeting the discharge criteria the patient was cleared to go home. 

## Discussion

CPVT is a rare genetic condition characterized by catecholamine-induced arrhythmias, which can lead to syncope and sudden death in children and young adults with morphologically normal hearts and normal baseline EKGs [7]. These patients present as a challenge to anesthesiologists as emotional and physical stressors can increase the risk of cardiac arrhythmias. Close monitoring and communication within the healthcare team is essential for the management of patients with CPVT. In this case report, we described the safe perioperative course that was executed in order to prevent arrhythmogenic complications in a patient with CPVT who underwent dental extractions under general anesthesia.

In order to minimize emotional stressors, preoperative and operating room healthcare personnel should be well-informed of the patient’s genetic disorder and perioperative plan. An experienced healthcare personnel or anesthesiologist should place an intravenous catheter to avoid the emotional stress of multiple attempts. This patient had minimal anxiety regarding intravenous catheter placement therefore no premedication was needed for IV placement. Premedication with midazolam prior to transport to the operating room, however, should be considered in patients with CPVT to reduce the risk of arrhythmia from catecholamine surge secondary to anxiety [8].

The anesthetic goals in a patient with CPVT include the avoidance of endogenous catecholamine surges secondary to fear or inadequate levels of anesthesia, avoidance of extrinsic catecholamines, especially from beta-adrenergic agents, and treatment of dysrhythmias should they occur [7]. According to Staikou et al., it is crucial to emphasize the necessity of not interrupting β-blocker therapy perioperatively in order to prevent CPVT symptoms from reoccurring [8]. A syncopal episode has been reported in a patient treated with nadolol after one missed dose [3]. Furthermore, cardiac defibrillator pads must be placed as a precaution for the development of malignant arrhythmias. As per Dornan et al., alternative options for dysrhythmias that did not self-terminate during exercise testing for CPVT patients include IV esmolol, IV magnesium, and defibrillation [4]. An ICD should only be considered in patients who have been resuscitated after cardiac arrest and who have experienced recurrent syncope or sustained ventricular tachycardia despite beta-blocker therapy [2,9]. Insertion of an ICD in patients with CPVT is not without risk as fatal ventricular storms can also be initiated through shocks [2].

The anesthetic plan should be individualized based on the patient’s level of anxiety, cooperation, and overall comorbidity. For example, general anesthesia was preferred over local anesthesia and sedation in our case due to the contraindication of epinephrine supplementation with local anesthesia and possible inadequate depth of anesthesia to maintain a calm state during the procedure. Similarly to previous reports, the anesthetics used to safely manage our patient with CPVT include fentanyl, propofol, rocuronium, sevoflurane, and dexmedetomidine infusion [7,10,11].

Avoidance of perioperative tachycardia is paramount as slight changes in the sympathetic activity in patients with CPVT can lead to arrhythmias. An intravenous beta blocker agent such as esmolol was carefully administered during the induction and intubation of our patient. Esmolol is an ideal agent as it is an ultra-short-acting cardioselective beta-1 adrenergic antagonist that can attenuate hemodynamic responses to perioperative noxious stimuli [7]. Dexmedetomidine is another useful agent for the management of patients with CPVT due to its sympatholytic properties. According to Matsumura et al., dexmedetomidine is expected to be effective for patients with increased adrenergic effects, and may also be effective in controlling agitation on emergence from general anesthesia [10].

Careful management of CPVT patients in the PACU is equally important as inadequate analgesia and postoperative nausea and vomiting are stressors that can induce cardiac arrhythmias. In our case, pain was adequately controlled through fentanyl administration, lidocaine injection, and dexmedetomidine infusion. As per Wang et al., dexmedetomidine for patients undergoing dental implantation can increase postoperative analgesia, reduce anxiety and inhibit sympathetic activity while minimizing respiratory impact [12]. Patients with dexmedetomidine infusion even at low doses such as 0.2 or 0.6 mcg/kg can achieve adequate sedation and analgesia without causing cardiorespiratory complications [12]. Additionally, ondansetron and dexamethasone are antiemetic agents that have been described to be safe in patients with arrhythmogenic disorders [9]. A study by Kenyon et al. demonstrated that ondansetron at a dose of 4 mg was safe in patients with long QT syndrome without adverse effects [13].

Literature on anesthetic management for patients with CPVT remains limited. Through our case report, we were able to describe a safe anesthetic practice for a CPVT patient who underwent an elective procedure. However, this anesthetic management may not be applied in different clinical situations. Further studies investigating how to manage CPVT patients in urgent or emergent surgeries are still needed as different clinical implications may change the course of the anesthetic plan and management. 

## Conclusions

In conclusion, catecholaminergic polymorphic ventricular tachycardia (CPVT) is a rare genetic condition characterized by catecholamine-induced arrhythmias, which can lead to syncope and sudden death especially in children and young adults. Successful perioperative management of CPVT patients requires a thorough understanding of the potential cardiac risks, careful planning throughout the perioperative period, close monitoring, and effective cooperation within the healthcare team.
